# Unraveling the immune microenvironment in primary CNS lymphoma

**DOI:** 10.1186/s40364-026-00945-9

**Published:** 2026-05-30

**Authors:** Sven Lorenzen, Vanja Zeremski, Mirjeta Berisha, Dimitrios Mougiakakos, Tobias R. Haage

**Affiliations:** 1 Department of Hematology, Oncology, Cell Therapy, and Radiation Therapy, Medical Center, Otto-von-Guericke University, Leipziger Str. 44, 39120 Magdeburg, Germany; 2 Magdeburg Center for Cell- and Immunotherapy, Otto-von-Guericke University, Magdeburg, Germany

**Keywords:** Primary CNS lymphoma, Immune microenvironment, T-cell immunity, Immune evasion, Prognostic scoring, Immunotherapy

## Abstract

Primary central nervous system lymphoma (PCNSL) is a rare malignant lymphoid condition that arises within the central nervous system, which is considered an immune-privileged site. Outcomes for patients with PCNSL have substantially improved over the past decade, largely due to treatment intensification. In recent years, it has become increasingly evident that the biology of PCNSL is not solely determined by the tumor itself, but also by its close and complex interaction with the immune microenvironment. Tumor-infiltrating lymphocytes, tumor-associated macrophages, and dendritic cells, in particular, reshape the immune milieu in PCNSL. Notably, an immunosuppressive microenvironment characterized by a high concentration of M2-like macrophages, a low concentration of CD8+ T-cells, and high expression of the cytokine IL-10 is associated with an unfavorable survival. Conversely, T-cell immunity and its abundance are pivotal for favorable treatment outcomes, highlighting the prognostic importance of immune competence within the central nervous system. The current standard of care for induction treatment is based on high-dose methotrexate as the central component, followed by consolidating high-dose chemotherapy and autologous stem cell transplantation in eligible patients. Long-term follow-up data from the IELSG32 and PRECISE trials demonstrated that up to 70% of patients remained alive at seven years, underscoring the curative potential of these strategies. Eligibility mainly depends on performance status, which is recognized as a key prognostic factor in standard risk stratifications. Nevertheless, relapse rates remain substantial, prompting growing interest in immunotherapeutic strategies. T-cell-based approaches, including among others checkpoint inhibitors, T-cell engagers, and CAR T-cells have achieved encouraging therapeutic success. However, the immune-privileged nature of the central nervous system poses immunological challenges that limit immune surveillance and facilitate tumor cell evasion, creating therapeutic obstacles in PCNSL. A comprehensive understanding of the manner in which alterations in oncogenic signaling influence immune evasion strategies holds considerable potential for enhancing future therapeutic approaches in PCNSL. Despite compelling evidence that the immune microenvironment significantly influences disease progression, established prognostic models for PCNSL do not yet consider inflammatory or immunological biomarkers. Given the significant prognostic and therapeutic implications of the immune microenvironment, its impact should be reflected and validated in future standard risk stratifications. Integrating validated immune biomarkers with emerging immunotherapeutic strategies has the potential not only to improve individual patient outcomes but also to optimize the overall structure of care for patients with PCNSL.

## Background

Primary central nervous system lymphoma (PCNSL) is a rare malignant lymphoid condition that is limited to structures of the central nervous system (CNS) without systemic involvement. The incidence is approximately 0.5 per 100,000 individuals per year, with a median age of 68 years and men being slightly more affected than women [1, 2]. Histopathological assessment is an integral part in the diagnosis of all malignant lymphomas including PCNSL. In accordance with the 5th edition of the World Health Organization (WHO) classification, primary diffuse large B-cell lymphoma (DLBCL) of the CNS is included within the entity of primary large B-cell lymphomas (LBCL) of immune-privileged sites. The distinction from systemic LBCL is based on the site of tumor manifestation [1, 3]. However, transcriptomic analyses allows clear discrimination between PCNSL and LBCL based on distinct translocation patterns and gene expression profiles. These analyses revealed recurrent mutations in Janus kinase/signal transducer and activator of transcription (JAK/STAT), nuclear factor ‘kappa-light-chain-enhancer’ of activated B-cells (NF-κB) and B-cell receptor (BCR) signaling pathways, including hallmark mutations *in myeloid differentiation primary response 88* (*MYD88*) L265P and *CD79B* [4, 5]. Moreover, together with testicular and vitreoretinal LBCL, PCNSL belongs to the group of LBCL arising in immune-privileged sites [3]. Indeed, the immune-privileged nature of the CNS leads to impaired immune surveillance and reduced efficacy of immune-based therapies, thereby facilitating tumor immune evasion and contributing to therapeutic challenges in PCNSL [6]. Outcomes for patients with PCNSL have substantially improved over the past decade, largely due to treatment intensification [7, 8]. The current standard of care for induction treatment is based on high-dose methotrexate (HD-MTX) as the central component, followed by consolidating high-dose chemotherapy and autologous stem cell transplantation (HDC-ASCT) [9–11]. This approach has resulted in durable disease control in a considerable proportion of patients. In the PRECIS trial, an intention-to-treat analysis of patients with PCNSL who achieved HDC-ASCT, revealed a two-year progression-free survival (PFS) rate of 70% and a two-year overall survival (OS) rate of 66% [10, 11]. Similar two-year PFS rates were reported in the IELSG32 trial [9]. A multicenter retrospective study of 174 immunocompetent patients with PCNSL undergoing intensified treatment regimens found that the two-year OS and PFS rates were 73.3% and 48.5%, respectively [12]. Long-term follow-up data from the IELSG32 and PRECISE trials further demonstrated that up to 70% of patients remained alive at seven years, underscoring the curative potential of HDC-ASCT-based treatment strategies [9, 10]. The improved outcome seems to be reserved for the HDC-ASCT-eligible patients. A meta-analysis, including elderly patients, showed that after a median follow-up period of 40 months the median OS and PFS for the entire cohort were 19 and 10 months, respectively [13]. A younger age (under 65 years) was significantly associated with favorable OS. This is also reflected in aforementioned clinical studies in which patients with PCNSL are eligible for intensive therapy. In accordance with current guidelines, whole-brain radiotherapy (WBRT) remains a viable alternative for fit patients with inadequate autologous stem cell harvest, as well as for those who decline to proceed with HDC–ASCT [14, 15]. In this context, current data support reduced-dose WBRT as consolidation after suitable induction treatment [1]. While initial treatment often leads to reasonable remission rates, the recurrence rate is high and recurrence treatment is challenging. Early recurrences of PCNSL typically occur within the first two years after initial diagnosis, resulting in poorer OS and PFS [16, 17]. 

In recent decades, the investigation of immunocompetence and T-cell immunity across malignancies, especially in various hematologic entities, has assumed a pivotal role. The immune-privileged nature of the CNS creates distinct immunological constraints that promote tumor cell evasion, particularly relevant for PCNSL, as this environment may adversely affect the efficacy of immune-based therapies [6]. So-called *immune-rich* subtypes of PCNSL are associated with a favorable prognosis, whereas an immunosuppressive microenvironment correlates with inferior outcomes [18, 19]. Although the immune microenvironment significantly influences disease progression, established prognostic models for PCNSL, such as the International Extranodal Lymphoma Study Group (IELSG) and Memorial Sloan-Kettering Cancer Center (MSKCC) models, do not consider inflammatory or immunological biomarkers [20, 21]. In several non-Hodgkin’s lymphoma (NHL) subentities besides PCNSL, including diffuse large B-cell lymphoma (DLBCL) and follicular lymphoma, lymphopenia was associated with poorer outcomes [22–24]. More recent risk models of PCNSL, including the three-factor (3F) score, incorporate lymphopenia as a marker for an impaired immune microenvironment in prognostic assessment [12, 25]. Unraveling the immune microenvironment in PCNSL is crucial for explaining its biological and clinical characteristics. Furthermore, this knowledge is essential for identifying therapeutic vulnerabilities that can be exploited by targeted and immunomodulatory therapies. The present review article aims to outline the biological and clinical impact of the immune microenvironment in PCNSL, with a particular view on myeloid cell and T-cell immunity as well as (oncogenic) signaling pathways in lymphomagenesis that drive the immunosuppressive phenotype. This review examines how the immune microenvironment is translated into a prognostic factor within the context of current PCNSL risk assessments and scoring systems, concluding with therapeutic implications and future directions of immunotherapies.

## Pathogenesis and tumor biology

### Immune cell composition of the microenvironment

**Fig. 1:**
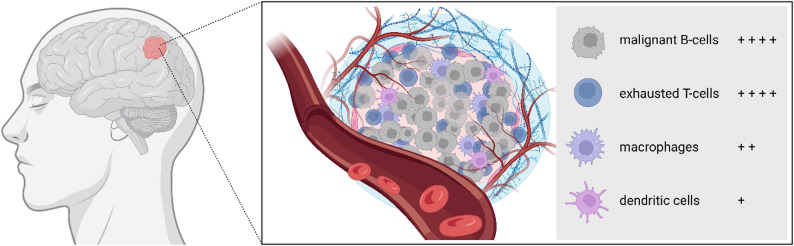
Composition of the immune microenvironment in PCNSL. Malignant B-cells almost completely displace the normal tissue, accompanied by the infiltration of effector cells (i.e., T-cells), professional antigen-presenting cells (i.e., dendritic cells), and innate myeloid immune cells (i.e., macrophages), collectively reshaping the immune milieu. Created with *Biorender.com* (https://BioRender.com/0r6gw83)

The tissue surrounding a tumor, which consists of various cell types (e.g., immune cells and connective tissue cells) and non-cellular components (e.g., extracellular matrix and signaling molecules), is referred to as the microenvironment and can directly and indirectly influence the tumor development, metastasis, and response to treatment. Wei et al. used single-cell ribonucleic acid (RNA) sequencing to examine tumor samples from PCNSL patients in comparison with normal human fetal brain tissue. In accordance with the tumor biology of PCNSL, B-cells were the most common cell population in the patient samples, along with T-cells, macrophages, and dendritic cells. In contrast, normal human fetal brain revealed a lack of peripheral immune cells and the presence of excitatory neurons and interneurons, radial glia, and inhibitory neuronal progenitor cells among the most common cell types [26]. Thus, malignant B-cells almost completely displace the normal tissue, a process accompanied by a pronounced immunological reaction characterized by the infiltration of effector cells (i.e., T-cells), professional antigen-presenting cells (i.e., dendritic cells), and innate myeloid immune cells (i.e., macrophages), collectively reshaping the immune milieu. Dendritic cells account for a high proportion of the immune microenvironment of PCNSL, with a similar distribution of so-called conventional, myeloid and plasmacytoid dendritic cells, as identified by their characteristic marker genes [26]. High-plex immunohistochemistry performed on tissue microarrays showed that besides CD68+ and CD163+ tumor-associated macrophages (TAMs) CD8+ T-cells are among the major components of the tumor microenvironment in PCNSL [27, 28]. An enrichment of CD8+ T-cells was particularly evident in the perivascular tissue, with a higher concentration than in the central tumor itself [28, 29]. However, Marcelis et al. described a remarkable intra- and intertumoral heterogeneity with a high variation for both subpopulations, macrophages and CD8+ T-cells. This heterogeneity was also identified in multi-omics analyses [18, 28]. Spatial transcriptomic analysis revealed distinct T-cell clusters in the PCNSL microenvironment including CD4+ naïve, CD4+ exhausted, effector memory T-cells as well as CD8+ naïve, CD8+ cytotoxic, CD8+ exhausted T-cells, and regulatory T-cells (Tregs) [30, 31]. Based on the immune microenvironment composition, especially T-cells with different degrees of activation and presence as tumor-infiltrating lymphocytes (TILs), *immune-rich* (“*immune-hot*”), *immune-intermediate*, and *immune-poor* subtypes can be distinguished. *Immune-rich* subtypes of PCNSL are characterized by a high density of activated CD8+ T-cells, macrophages and Tregs [18, 19]. TAMs, as an integral component of the immune microenvironment in PCNSL, are usually dichotomized into two subsets: classically activated, pro-inflammatory, tumoricidal (i.e., M1-like, CD68+ CD163low) and alternatively activated, tumor-promoting (i.e., M2-like, CD68+ CD163high) macrophages, respectively [28, 32, 33]. M2-like macrophages are known to release immunosuppressive cytokines, thereby indirectly promoting tumor growth. Malignant B-cells actively shape the tumor microenvironment by promoting the differentiation of monocytes into anti-inflammatory macrophages. Under the influence of B-cell-derived γ-aminobutyric acid (GABA), monocytes purified from bone marrow cells differentiated into anti-inflammatory, interleukin-10 (IL-10)-secreting macrophages, as demonstrated by Zhang et al. [34] In comparison with the microenvironment of primary testicular lymphoma, PCNSL has been shown to exhibit lower concentrations of TILs, a higher T-cell exhaustion index and a predominant presence of CD163+ TAMs [35]. Recent reviews by Hernández-Verdin et al., Jin et al., and Zhong et al. examined the role of the tumor microenvironment in inducing immunosuppression and driving lymphomagenesis in PCNSL [36–38]. Fig. 1 outlines the immune microenvironment composition in PCNSL.

Takei et al. investigated the influence of alpha-smooth muscle actin (αSMA)-positive spindle cells as non-immune cellular components of the tumor microenvironment in the course of a histopathological assessment. Extratumoral expression of αSMA was significantly associated with an infiltration of CD3+/CD4+ TILs and CD68+ TAMs [39]. The examination of non-immune cellular components of the tumor microenvironment, as in this case cancer-associated fibroblasts, can thus be used to assess its immunological composition. Furthermore, it has been demonstrated that non-immune cellular components, such as endothelial cells, pericytes, and hypertrophic astrocytes, support angiogenesis and proliferation of malignant B-cells potentially through the expression of nestin and the octamer-binding transcription factor 4 (OCT4) [40]. 

### Mechanisms of immune evasion in PCNSL

Immune checkpoint receptors such as lymphocyte-activation gene 3 (LAG-3), programmed cell death protein 1 (PD-1, CD279) and T-cell immunoglobulin and mucin-domain containing-3 (TIM-3) particularly expressed by T-cells are crucial in shaping immune responses and immunity in PCNSL [41]. 

Their presence is associated with T-cell dysfunction and they are recognized as exhaustion markers. Examinations of biopsy-derived T-cells revealed an evident exhaustion signature with an upregulation of canonical exhaustion molecules, including LAG-3, PD-1, and TIM-3, as compared to peripheral blood-derived T-cells [31]. An examination by Seguita et al. demonstrated that PCNSL-TILs exhibited immunohistochemical positivity for PD-1 and, to a lesser extent, for the transcription factor forkhead box P3 (FOXP3), which is typically expressed by Tregs [42]. The analysis of 53 PCNSL samples by Sethi et al. revealed PD-1 expression in 98% of all TILs [43]. An upregulation of LAG-3 and TIM-3 was noticeable in CD8+ T-cells with a high PD-1 co-expression [28]. Liu et al. examined the correlation of PD-1 with CD4+ or CD8+ TILs in a cohort of 52 PCNSL patients. Remarkably, the expression of PD-1 was significantly associated with infiltrating CD8+ T-cells, but not CD4+ T-cells [44]. This selective association of PD-1 with CD8+ TILs in PCNSL points to functionally constrained cytotoxic immunity in the absence of effective CD4+ T-cell assistance.

TAMs have been shown to express both, PD-1 and programmed death-ligand 1 (PD-L1, CD274) [45–47]. In chronic lymphocytic leukemia (CLL), it has been demonstrated that PD-1 stimulation can result in immune metabolic dysfunctions within monocytes, including phagocytosis and Bruton’s tyrosine kinase (BTK) signaling. Conversely, the disruption of PD-1/PD-L1 signaling has been shown to reverse these dysfunctions [48]. In a comprehensive study of murine and human TAMs in the (non-lymphoid) context of colorectal cancer, PD-1 expression negatively influenced their phagocytic potency against tumor cells. Interestingly, PD-1 expression was significantly higher on M2- as compared to M1-like TAMs and the frequency of PD-1+ TAMs within the M2 subpopulation increased with disease stage [46]. In PCNSL patients, the immune functions of TAMs are significantly suppressed as compared to normal human fetal brain counterparts [26]. Certain studies described the expression of PD-L1 on TAMs in patients with PCNSL [42, 43, 49]. In the study of Sun et al., the range of PD-L1-positive TAMs was highly variable, with a median proportion of 36.1%. Furthermore, PCNSL patients with a high M2/M1 ratio tended to have a higher proportion of TAMs expressing PD-L1, which is consistent with the characteristic preferential expression of PD-L1 by M2-polarized macrophages [49]. A murine orthotopic xenograft model using primary PCNSL cells revealed that, in accordance with the aforementioned patient-derived data, PD-1-expressing TAMs were predominantly M2-like [32]. As suggested by Jiménez et al., upregulation of PD-1 is mediated by direct interaction of M2-like macrophages with malignant B-cells. It has been demonstrated that PD-1/PD-L1 impairs the phagocytic capacity of TAMs and inhibits M1 polarization, thereby compromising their tumoricidal functions [46, 50]. In addition to PD-1/PD-L1-mediated immune suppression, other regulatory mechanisms involving myeloid cells have been identified. These include upregulation of signal regulatory protein α (SIRPα), the receptor for the CD47 “don’t-eat-me” signal, which further impairs macrophage phagocytic capacity, as well as co-expression of PD-1 and TIM-3 on the same macrophage populations, both acting as inhibitory immune checkpoints that compromise myeloid effector functions [28, 32]. Moreover, TAMs actively suppress bystander immune cells through the release of immunosuppressive cytokines, such as IL-10 and TGF-β, thereby inhibiting effector T-cell functions and promoting the induction of Tregs. Collectively, these mechanisms reinforce a tolerogenic tumor microenvironment (as reviewed by Pan et al. and Wang et al.) [33, 51]. In summary, both the abundance and functional state of TAMs play a pivotal role in the PCNSL microenvironment, particularly through their dynamic interplay with TILs. This myeloid-driven immune regulation influences tumor progression, intratumoral heterogeneity, and response to therapy, supporting the notion of a myeloid immunosuppressive barrier in PCNSL.

Malignant B-cells, which predominantly exhibit an activated phenotype, contribute both directly and indirectly to the immunosuppressive tumor microenvironment. They directly inhibit CD8+ effector T-cell functions through the secretion of immunosuppressive mediators such as IL-10, promote the induction of Tregs and skew monocyte differentiation towards an immunosuppressive, M2-like macrophage phenotype [34]. Beyond the upregulation of immune checkpoint receptors on infiltrating immune cells, malignant B-cells in PCNSL display strong expression of the corresponding immune checkpoint ligands. This enables direct inhibitory crosstalk with receptor-high TILs, thereby promoting T-cell dysfunction and exhaustion. Accordingly, spatial transcriptomic analyses revealed that clusters of highly malignant B-cells are surrounded by regions enriched for exhausted T-cell signatures, reflecting localized zones of profound immunosuppression [31]. In PCNSL, upregulation of PD-L1 and PD-L2 (CD273) is also genetically driven by copy number gains of the 9p24.1 locus (9p24.1/*PD-L1*/*PD-L2*), thereby reinforcing immune evasion through enhanced immune checkpoint ligand expression [52–54]. However, the significance of 9p24.1/*PD-L1*/*PD-L2* is controversially discussed in the literature. While Epstein-Barr virus (EBV)-positive PCNSL undoubtedly showed pronounced PD-L1 expression, amongst others driven by activated JAK/STAT signaling, 9p24.1/*PD-L1/PD-L2* copy number alterations were barely evident in some studies, partially within an EBV-negative status as far as known [43, 53, 55]. In the study by Ou et al., a lack of PD-L1 expression was detected in about one third of PCNSL. However, no information on EBV status was provided, thereby underscoring the importance of comprehensive diagnostic procedures [56]. 

**Fig. 2:**
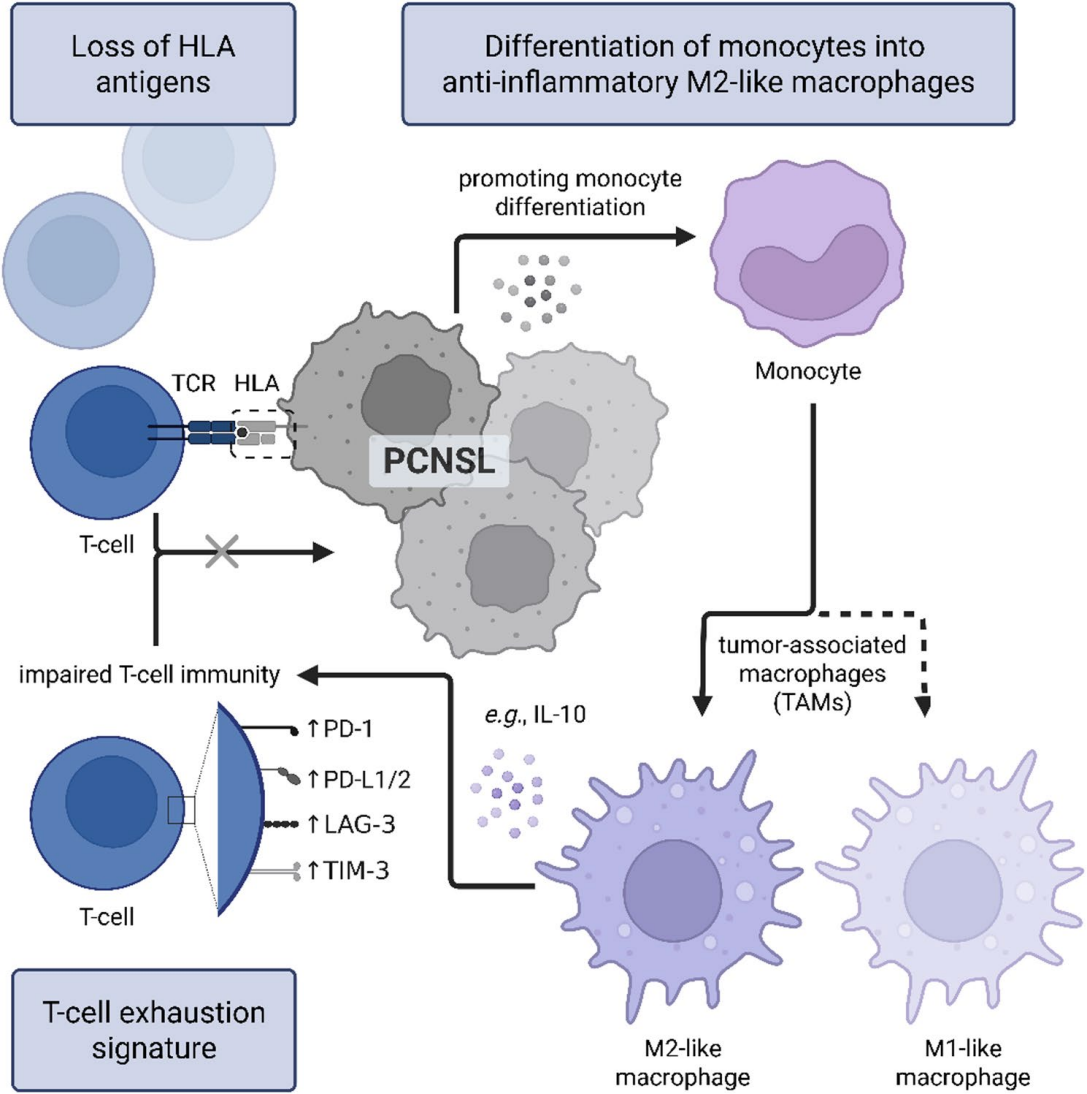
Overview of immune evasion strategies in PCNSL. Malignant B-cells, in particular, contribute to the immunosuppressive tumor microenvironment by skewing monocyte differentiation towards a M2-like macrophage phenotype. This overview is not intended to be comprehensive. Created with *Biorender.com* (https://BioRender.com/t2xn8wy)

In PCNSL, malignant B-cells can also evade immune surveillance through the loss of human leukocyte antigen (HLA) class I and II expression, thereby rendering malignant B-cells functionally “invisible” to the adaptive immune system [52–54]. Mindermann et al. reported HLA class I loss with a prevalence of 64%, HLA class II loss with a prevalence of 59% in PCNSL, respectively [53]. Notably, down-modulation of HLA class I was associated with an *immune-poor* PCNSL subtype characterized by absent or minimal T-cell activation [19]. In parallel, the scarcity of natural killer (NK) cells within the PCNSL microenvironment limits effective “missing-self”–driven immune rejection, further reinforcing immune escape. At the genomic level, large PCNSL cohorts frequently exhibit chromosomal losses at 6p or 6q and focal homozygous deletions at 6p21.3, encompassing the HLA class I and II gene loci [57, 58]. Recurrent segmental alterations affecting chromosome 6p suggest that HLA loss is a genetically imprinted feature of PCNSL. Moreover, unsupervised bihierarchical clustering analyses have revealed pronounced genomic instability in the majority of PCNSL cases, reflected by a high overall burden of copy number alterations and frequent loss of tumor suppressor genes such as *CDKN2A* and/or *FHIT* [52]. Collectively, these mechanisms converge to establish a highly effective immune evasion strategy in PCNSL, combining impaired antigen presentation with limited innate immune compensation, as illustrated in Fig. 2.

### Oncogenic signaling as a link between lymphomagenesis and immune evasion

The tumor microenvironment of PCNSL is shaped by a dynamic interplay between oncogenic signaling programs driven by genetic alterations and immune-regulatory mechanisms. In addition to sustaining the survival and proliferation of malignant B-cells, oncogenic pathways actively contribute to immune evasion by fostering an immunosuppressive microenvironment. In this context, the communication between lymphoma cells and myeloid populations via cytokines is particularly relevant, as the secretion of IL-10 by anti-inflammatory macrophages is capable to suppress CD8+ T-cell functions [34, 59]. Genetic alterations affecting BCR and toll-like receptor (TLR) signaling are central to these processes [60]. The most frequent mutations involve the *MYD88* gene, a key adaptor molecule of TLR and IL-1 receptor signaling [56, 61, 62]. Activating *MYD88* mutations, most notably *MYD88*-L265P, induce constitutive downstream signaling through the MYD88/interleukin-1 receptor-associated kinases (IRAK) complex [63]. These mutations are often accompanied by mutations in *CD79B* and *CARD11* [52, 56, 64]. The co-occurrence of *MYD88* and *CD79B* mutations reflects the activation of both BCR- and TLR-dependent signaling cascades and constitutes a hallmark of the PCNSL transcriptional landscape [52, 65, 66]. Genomic profiling consistently places PCNSL close to the *MYD88*/*CD79B*-mutated (MCD/C5) subtype of DLBCL [4, 67–70]. This subtype is associated with poor clinical outcomes and is believed to originate from long-lived memory B-cells [4]. Current molecular classification systems, including the LymphGen algorithm and the recently published neural network–based probabilistic classifier DLBclass, facilitate the identification of distinct genetic subtypes of DLBCL. Herewith, PCNSL is assigned to the MCD subtype (LymphGen) or the C5 cluster (DLBclass) [67, 71]. Importantly, oncogenic MYD88 signaling has effects that extend beyond cell-intrinsic growth advantages to directly affect immune regulation. *MYD88*-L265P is closely associated with the constitutive activation of the NF-κB and JAK/STAT signaling pathways, which promote immune evasion [61]. A longitudinal analysis of mice harboring a *MYD88*-L265P mutation revealed that continuous MYD88 activation is associated with the clonal transformation of differentiating B-cells and progressive peripheral B-cell expansion [72]. In lymphoma cells with deregulated NF-κB signaling, *MYD88*-L265P has been shown to induce the upregulation of immune checkpoint ligands PD-L1 and PD-L2 [73]. This provides a mechanistic link between oncogenic signaling and T-cell inhibition. Consistent with this concept, aberrant JAK/STAT signaling has been demonstrated to induce PD-1 ligand expression in other B-cell lymphomas, supporting the functional relevance of this axis in PCNSL [74, 75]. Additional oncogenic alterations further reinforce immune evasion. A significant co-occurrence of *MYD88* and *TBL1XR1* mutations was identified in PCNSL, with *TBL1XR1* mutations present in around 40% of cases [4]. As analyzed by Venturutti et al., *TBL1XR1* mutations drive lymphomagenesis through the generation of abnormal immature memory B-cells [76]. Mutations affecting the phosphatidylinositol 3-kinase (PI3K)/AKT/mechanistic target of rapamycin (mTOR) pathway, which are frequently observed in PCNSL, converge on NF-κB signaling networks [65, 77]. These mutations are also known to modulate cytokine production, metabolic fitness, and immune checkpoint expression. *MYD88*-driven lymphomas also exhibit reduced dependence on co-stimulatory T-cell signals [78]. This suggests that malignant B-cells progressively acquire functional autonomy from adaptive immune surveillance during lymphomagenesis. Viral status further modulates the oncogenic–immunologic interface. EBV-positive PCNSL cases typically lack *MYD88*-L265P mutations and instead exhibit aberrant somatic hypermutations and frequent *SOCS1* alterations, which can lead to JAK/STAT signaling disinhibition [79, 80]. Transcriptomic and epigenetic profiling has revealed that EBV-negative PCNSL exhibits enhanced BCR and Wnt/β-catenin signaling with correspondingly increased spleen tyrosine kinase (SYK) activity. In contrast, EBV-positive tumors are enriched for IL-10 and Notch signaling pathways, highlighting distinct yet convergent routes toward immunosuppression [81]. 

**Fig. 3:**
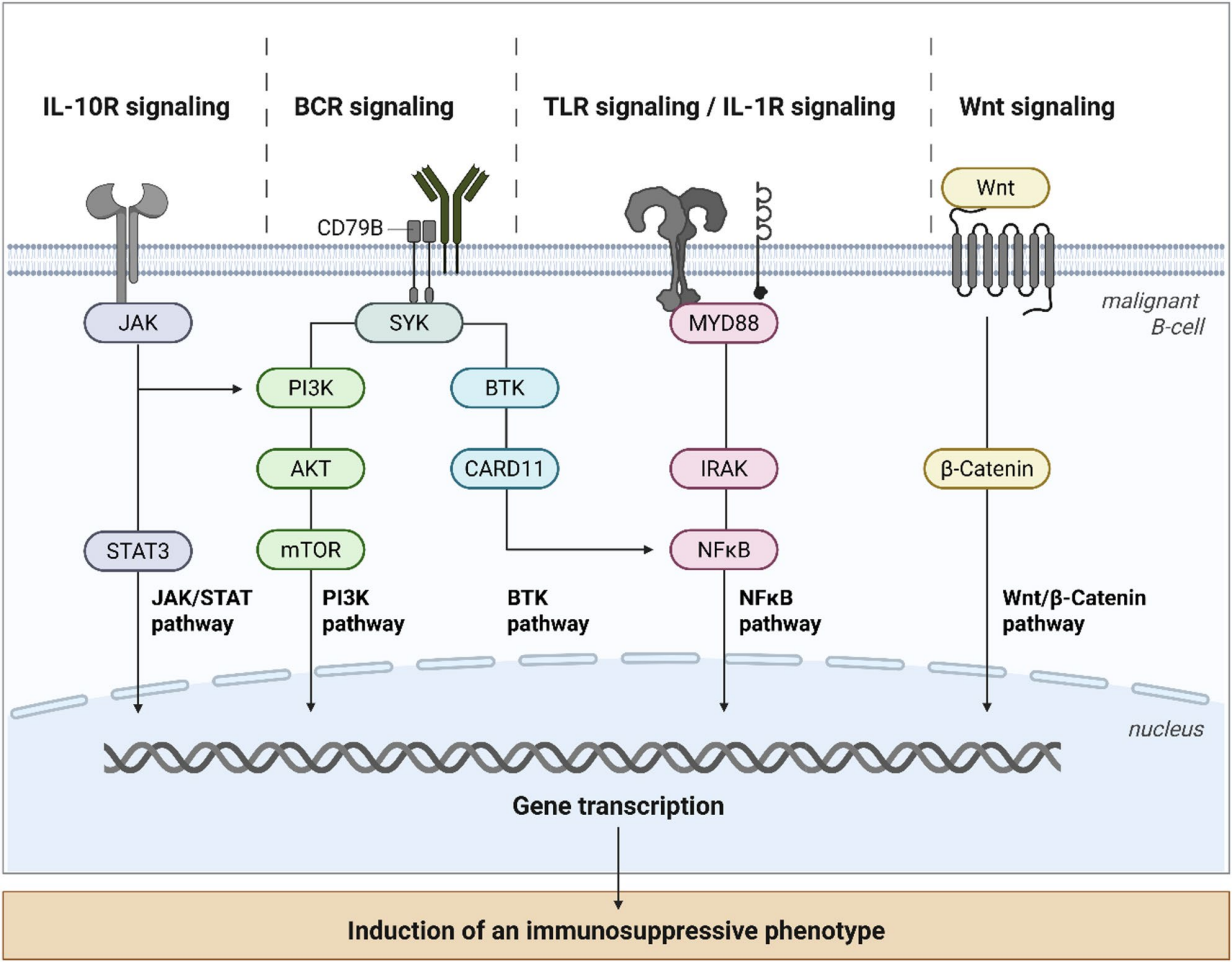
Schematic overview of signaling pathways in PCNSL. The crosstalk between oncogenic signaling and immunosuppressive mechanisms may promote an immunosuppressive phenotype in PCNSL. This overview is not intended to be comprehensive. Created with *Biorender.com* (https://BioRender.com/scdeikk)

Collectively, these data indicate that oncogenic signaling pathways in PCNSL not only drive malignant transformation but also actively sculpt an immunosuppressive tumor microenvironment through checkpoint induction, cytokine signaling, and reduced reliance on T-cell–mediated control. Important signaling pathways in PCNSL are depicted in Fig. 3. A comprehensive understanding of how genetically encoded signaling programs promote immune evasion will be critical for the rational development of targeted and immune-modulatory therapeutic strategies in PCNSL.

## Prognostic factors and scoring systems: impact of the immune microenvironment

### Influence of myeloid cells, lymphocytes, and immune checkpoints

It is not unexpected that certain genomic alterations (including *BTG1*, *ETV6*, *KMT2D*, and *TP53* mutations) that promote uncontrolled proliferation, epigenetic deregulation, impaired differentiation, and genomic instability lead to an unfavorable prognosis [57, 58]. However, beyond tumor-intrinsic genomic features, the immune microenvironment emerges as a critical determinant for outcome. In a large cohort of immunocompetent patients with PCNSL, the presence of reactive perivascular T-cell infiltration as a histopathological parameter was confirmed as favorable [82]. A high density of especially CD8+ T-cells in both, the tumor core or perivascular niche, was independently shown to be associated with a better OS [18, 27, 28, 83]. Roemer et al. used a multi-scale computational framework approach to investigate how the tumor microenvironment influences the survival of PCNSL patients. Combining computational parameters of cellular frequency, architectural and spatial interaction of 1,393 multiplex imaging data generated from 88 patient samples, different microenvironmental prognostic subgroups were identified. Indeed, this analysis confirmed that both perivascular and intratumoral T-cell infiltration represent favorable indicators for survival [54]. In a small North Indian cohort of overall 44 PCNSL patients, however, a high concentration of CD4+ TILs was associated with prolonged OS and PFS, whereas CD8+ TILs were not correlating with survival parameters [84]. In this context, PD-L1 expression on immune cells may reflect an interferon-γ (IFN-γ)-driven, activated immune microenvironment rather than purely immunosuppression, serving as a surrogate marker of ongoing T-cell activity. Notably, CD4+ T-cells may exert a cooperative helper function by supporting antigen presentation, sustaining effector T-cell responses, and orchestrating broader immune activation. Importantly, these findings need to be interpreted with caution, as regional and cohort-specific factors, including genetic background, treatment regimens, EBV prevalence, and methodological differences in immune profiling, may substantially contribute to divergent observations across studies and should be carefully considered when drawing general conclusions. In line with this, infiltration with CD4+ or CD8+ TILs and the expression of exhaustion markers do not necessarily correspond statistically significant with established clinicopathologic risk factors [44, 85]. As in other entities, one factor that is still underrepresented is the sex-specific influence on disease. For example, expression of FOXP3 as the prototypical transcription factor of Tregs was associated with poorer OS only in female PCNSL patients [86]. 

As demonstrated, high levels of PD-L1 and PD-L2 on leukocytes within the tumor microenvironment can be associated with superior survival [27, 87]. The infiltration of CD8+ TILs or CD68+ TAMs correlated positively with PD-L1 expression [83, 88]. In line with the finding that PD-L1 expression is enriched in CD8+ T-cells, patients with PCNSL and low tumoral CD8+ T-cell density exhibit low PD-L1 expression in the tumor microenvironment [44]. Again, the PD-L1 expression on TILs and TAMs and its influence on prognosis still remains controversial [49, 89]. The role of PD-L1 is highly context-dependent and may reflect either immune activation, acting as an IFN-γ-induced marker on TAMs and T-cells, or active immunosuppression through engagement of its cognate receptors, with the net biological effect determined by timing, cellular composition, and the functional state of the tumor microenvironment. In a study of Sun et al., TAMs with strong PD-L1 expression were associated with an inferior OS and PFS. In addition, a shift from M1- towards M2-like TAMs, which typically express higher levels of PD-L1, is indicative of unfavorable prognosis and is also observed in DLBCL [49, 90]. Consequently, Marcelis et al. showed that a high M1/M2 ratio is associated with a favorable outcome [28]. In a preclinical murine PCNSL model assessing CD19-directed chimeric antigen receptor (CAR) T-cell therapy, mice that relapsed exhibited a marked skewing of TAMs toward an M2-like phenotype, highlighting macrophage-mediated immune adaptation as a potential mechanism of resistance [91]. Several studies underline the context-dependent prognostic relevance of PD-L1 expression on TAMs in PCNSL. Sun et al. demonstrated that combining PD-L1 expression with the M2/M1 macrophage ratio improves prognostic stratification, with the poorest outcomes observed in patients exhibiting both, high PD-L1 expression on macrophages and a high M2/M1 ratio [49]. In contrast, Furuse et al. and Roemer et al. reported improved OS in association with high PD-L1 expression on TAMs or peritumoral macrophages [54, 92]. Importantly, Roemer et al. further showed that an overall abundance of TAMs in conjunction with low T-cell infiltration was associated with unfavorable outcome, underscoring that PD-L1 expression on macrophages may reflect either immune activation or immune suppression depending on the broader cellular context [54]. Interestingly, increased infiltration by activated glial cells in PCNSL has been associated with enhanced accumulation of M2-like TAMs and collectively linked to an increased relapse risk [93]. This observation suggests that glial cells may act as key immunoregulatory elements within the CNS tumor microenvironment, both through the release of immunomodulatory cytokines and by serving as a local reservoir for TAMs via resident microglia, in addition to recruited peripheral monocytes. Tumor infiltration with CD163+ M2-like TAMs significantly correlates with cerebrospinal fluid (CSF) IL-10. Other established clinicopathological risk factors such as age (<60 vs. >60 years) or Eastern Cooperative Oncology Group performance status (ECOG PS 0–2 vs. 3–4) did not correlate with TAMs infiltration [94]. Although, overall expression of PD-L1 within the tumor microenvironment is not necessarily of predictive potency, the concentration of soluble sPD-L1 and sPD-L2 within the CSF could add valuable diagnostic and prognostic information [43, 55, 95]. In a small cohort of 46 PCNSL patients, high levels of CSF sPD-L1 were associated with an unfavorable OS [95]. 

**Fig. 4:**
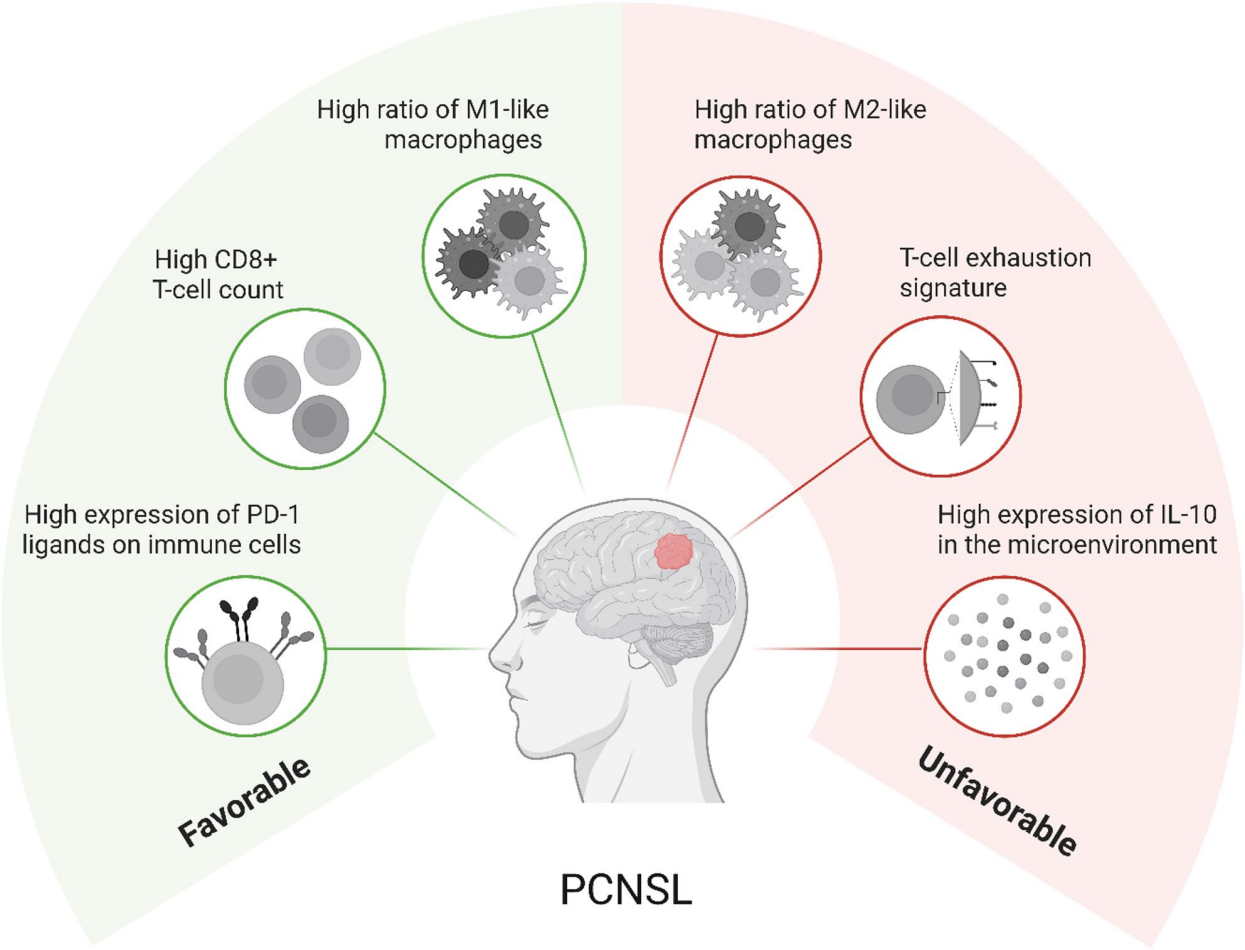
Influence of the immune microenvironment on prognosis in PCNSL. An increased ratio of classically activated, pro-inflammatory, tumoricidal M1-like macrophages as well as a high expression of PD-1 ligands on immune cells, and a high CD8+ T-cell count are associated with a favorable outcome. Conversely, an elevated ratio of alternatively activated, tumor-promoting M2-like macrophages, T-cell exhaustion, and high expression of the immunosuppressive cytokine IL-10 are indicative for an unfavorable outcome. Created with *Biorender.com* (https://BioRender.com/tzw8ki2)

In addition, advances in omics-based technologies have substantially expanded the repertoire of prognostically relevant biomarkers in PCNSL, enabling more detailed characterization of the disease in an immune context beyond conventional histopathology. Notably, CSF proteomic signatures have been shown to distinguish PCNSL from non-lymphoid brain tumors and healthy donors, underscoring their diagnostic and biological relevance [96]. At the molecular level, the presence of *MYD88*-L265P in conjunction with STAT3 activation, which is linked to immunosuppressive myeloid phenotypes, has been consistently associated with inferior clinical outcome [97, 98]. In primary PCNSL samples, STAT3 positivity and immunohistochemical IL-10 expression were predominantly observed in patients with multifocal disease, further linking oncogenic signaling to an immunosuppressive microenvironment [98]. Concordantly, elevated baseline CSF IL-10 levels (>34.48 pg/mL) correlated with unfavorable PFS and might reflect macrophage-driven immune regulation as TAMs infiltration and CSF IL-10 positively correlate [99]. In contrast, *CD79B* mutations were associated with improved PFS and OS compared with *CD79B*-unmutated PCNSL, suggesting that distinct B-cell-intrinsic signaling alterations may differentially shape immune interactions [55]. Taken together, these findings highlight that clinical outcome in PCNSL is determined by a complex interplay of genetic alterations, soluble immune mediators, and cellular immune composition, emphasizing the pivotal role of the immune microenvironment in disease progression and prognosis (see Fig. 4).

### Current prognostic assessment in PCNSL and future directions

The application of prognostic factors and scoring systems is generally conducive to the risk-stratification of patients and the selection of suitable therapeutic interventions. Two widely used scoring systems for risk stratification of patients with newly diagnosed PCNSL are the IELSG and MSKCC prognostic models [20, 21, 100]. The IELSG developed a prognostic scoring system that defines three independent risk groups based on the following five factors: age >60 years, ECOG PS ≥2, elevated lactate dehydrogenase (LDH) serum level, elevated CSF protein concentration, and involvement of deep brain structures [21]. The survival outcome was analyzed in an international multicenter retrospective study involving 378 immunocompetent patients with PCNSL, of whom the IELSG prognostic score could be examined in 105 patients. The resulting IELSG score was able to distinguish three different risk groups depending on the number of features mentioned: low risk (0 to 1 features), intermediate risk (2 to 3 features), and high risk (4 to 5 features). Herewith, the two-year OS was significantly distinguished between the risk groups (80%±8% vs. 48%±7% vs. 15%±7%) [21]. The MSKCC prognostic model is predicated on the assumption that age and Karnofsky performance status (KPS) are the variables that have been consistently and independently identified as prognostic factors [20]. These findings have led to the identification of three distinct risk groups: low risk (age <50 years and KPS ≥70), intermediate risk (age ≥50 years and KPS ≥70) and high risk (age ≥50 years and KPS <70) [20]. The MKSCC score could distinguish between patient cohorts in terms of median OS and event-free survival (EFS), depending on the risk group (median OS: 8.5 vs. 3.2 vs. 1.1 years, median EFS: 2.0 vs. 1.8 vs. 0.6 years) [20]. Lymphopenia was associated with worse outcomes in several NHL subentities [22–24]. Therefore, Jang et al. analyzed prognostic factors, including the absolute lymphocyte count (ALC), in 81 patients with PCNSL receiving HD-MTX-based therapy [25]. Receiver operating characteristic analysis defined lymphopenia with an ALC cut-off of ≤875 × 10^6^/L as significant in the discrimination of survival outcomes. Lymphopenia alone, possibly indicating a decreased anti-tumor immunity in PCNSL, was associated with an unfavorable five-year PFS (10.9% vs. 43.9%) and five-year OS (22.3% vs. 58.5%) [25]. In combination with previously identified unfavorable features (ECOG PS >1 and age >50 years), lymphopenia (ALC ≤875 × 10^6^/L) was included in a so-called three-factor (3F) score, which delineated three different risk groups depending on the number of factors mentioned: low risk (1 factor), intermediate risk (2 factors), and high risk (3 factors). The 3F score could distinguish between the low/intermediate and high-risk groups in terms of five-year PFS (42.3%/43.7% vs. 10.1%) and five-year OS (64.0%/63.0% vs. 11.7%) [25]. A multicenter, retrospective analysis of altogether 174 newly diagnosed PCNSL patients undergoing intensified treatment regimens identified the 3F score as stratifying the widest prognostic spectrum compared to the IELSG and MSKCC scores [12]. Table 1 provides a concise overview of the variables employed in the IELSG, MSKCC and 3F scores.

**Table 1:** Comparison of variables in the IELSG, MSKCC, and 3F prognostic scores for PCNSL

Variables	IELSG score [21]	MSKCC score [20]	3F score [25]
Age	>60 years	≥50 years	>50 years
Performance status	ECOG PS ≥2	KPS <70	ECOG PS >1
Elevated LDH	yes	-	-
Elevated CSF protein	yes	-	-
Involvement of deep brain structures	yes	-	-
Lymphopenia	-	-	ALC ≤875 × 10^6^/L
Low risk	0–1 points	0 points	1 point
Intermediate risk	2–3 points	Age ≥ 50 years and KPS ≥ 70	2 points
High risk	4–5 points	2 points	3 points

Abbreviations: 3F score - Three-factor score, ALC - Absolute lymphocyte count, CSF - Cerebrospinal fluid, ECOG - Eastern Cooperative Oncology Group, IELSG - International Extranodal Lymphoma Study Group, KPS - Karnofsky performance status, LDH - Lactate dehydrogenase, MSKCC - Memorial Sloan-Kettering Cancer Center, PS - Performance status

More recent assessments aim to refine prognostic accuracy in PCNSL by integrating so-called independent predictors of survival that are not fully represented in existing models. Although these scores often require further validation in prospective or multicenter approaches, they provide insight into how prognostic assessment can be reconsidered. The prognostic nutritional index (PNI), for example, a score first described decades ago in a surgical setting, combines the influence of serum albumin concentration and ALC. The combination of hypoalbuminemia and lymphopenia, representing a low PNI score, has been shown to be associated with an unfavorable prognostic outcome, a finding that has been validated in hematological diseases, particularly various NHL entities [101, 102]. In patients with PCNSL, a low PNI score was predictive of poor PFS [103]. Hypoalbuminemia and lymphopenia were also individually identified as unfavorable prognostic factors in patients diagnosed with DLBCL or PCNSL [22, 23, 104–106]. As described by Wei et al., a prognostic model based on serum albumin and ECOG PS was able to stratify different risk groups in PCNSL based on five-year OS rates [107]. Other risk factors analyzed for PCNSL cohorts include, among others, LDH-to-lymphocyte ratio, LDH-to-monocyte ratio, neutrophil-to-lymphocyte ratio, platelet-to-lymphocyte ratio, the pan-immune-inflammation value or systemic inflammation indices [107–114]. Recently, the International Primary CNS Lymphoma Collaborative Group (IPCG) published a prognostic stratification scheme specifically designed for the assessment of immunodeficiency-associated PCNSL [115]. The IPCG prognostic score uses the variables age >60 years, KPS <70, and EBV positivity to distinguish between low risk (1 factor), intermediate risk (2 factors), and high risk (3 factors) (median OS: 135 vs. 29 vs. 3 months). Thus, the IPCG score builds on the foundation of age and performance status already recognized by the IELSG, MSKCC and 3F scores, but again does not incorporate inflammatory or immunological biomarkers.

As it is limited to structures of the CNS, obtaining sufficient and representative primary tumor material for histopathological processing is challenging in PCNSL. Immunophenotyping of peripheral lymphocyte subsets could thus be an adequate alternative for monitoring and modelling prognosis [41]. Berthelot et al. conducted a lymphocyte immunophenotyping analysis on a cohort of 52 patients with newly diagnosed PCNSL [116]. Lymphopenia (affecting B-cells, T-cells or NK cells) was detected in 64% of cases. A high CD4+/CD8+ ratio was associated with poor PFS and tended to correspond with poorer OS and a therapy-refractory state, explicable by reduced anti-tumor activity of CD8+ T-cells [116]. In a cohort of overall 39 PCNSL patients, the clinical significance of Th1/Th2/Th17 cytokines and lymphocyte subsets in peripheral blood was examined [117]. Compared to patients with DLBCL or healthy donors, PCNSL patients had a higher CD4+/CD8+ ratio. Following treatment, an overall increase in CD8+ T-cells and a decrease in the CD4+/CD8+ ratio were observed [117]. Beyond the influence of T-cell immunity on prognosis, Lin et al. investigated the impact of peripheral NK cell counts on prognosis [118]. Among all NK cell analyses of overall 161 PCNSL patients, the median NK cell count was 198/µL (13–1890/µL). Patients who responded to treatment, as defined by reaching partial or complete remission, had a higher median NK cell count than non-responding patients with PCNSL. A trend towards better PFS and a significantly improved OS was shown by an NK cell count >165/µl [118]. However, the opposite effect on the OS has also been reported in cases of increased NK cell counts [100]. Again, these findings need to be interpreted with caution and with regard to regional and cohort-specific factors in immune profiling that may contribute to divergent observations. Furthermore, systemic biomarkers derived from peripheral blood may not accurately reflect the local microenvironment of PCNSL, while stereotactic biopsy specimens may not completely capture spatial heterogeneity. A promising approach is to bridge the gap between the tumor microenvironment and the systemic response of patients by measuring biomarkers in CSF. CSF-based detection of *MYD88* mutations and IL-10 levels demonstrates high sensitivity and specificity for identifying PCNSL at both initial diagnosis and relapse [119–121]. The evaluation of CSF samples has the potential to contribute to the development of less invasive and effective diagnostic and prognostic methods in PCNSL by capturing CNS-specific immune and tumor-associated signals.

In view of the impact of genetic alterations on outcomes in PCNSL, these markers are now also being considered in the development of prognostic models. As already considered for DLBCL, the dual expression of B-cell lymphoma 2 (BCL-2) and MYC is linked to an unfavorable outcome and a higher risk of CNS relapse [122]. In PCNSL, the co-expression of BCL-2 and MYC was consistent with a lower response rate to induction therapy and a 13-fold higher risk of progression within five years [123]. Geng et al. further identified high-risk aberrations associated with an early lymphoma progression: focal 6p21.3 homozygous deletions, 6p copy-neutral loss of heterozygosity as well as *BTG1*, *ETV6*, and *TP53* mutations [58]. Patients with at least one high-risk alteration had a higher risk of progression despite methotrexate (MTX)-based induction or dose-intensive consolidation therapy. Awareness of this high-risk state justifies an appropriate level of surveillance. In addition, in-vitro single-cell transcriptome analysis could unveil heterogenic gene expression in MTX-resistant PCNSL [124]. Machine learning-based models can provide prognostic predictions of MTX-based treatment for PCNSL using lipidomics as a biomarker analysis from CSF [125]. Takashima et al. examined the influence of microRNA (miRNA) on prognosis prediction in PCNSL [126]. miRNAs are short non-coding strands of RNA that exert an influence on gene regulation. It has been shown that miRNA (*miR-21*, *miR-19*, *miR-92a*) in CSF can be used for early identification and diagnostic evidence of PCNSL [127, 128]. Although there are differences in the exact type of miRNA, PCNSL show different miRNA expressions compared to DLBCL, emphasizing the different molecular nature of both entities [129]. Furthermore, the prognosis for PCNSL could be impacted by the homeostasis of non-cellular components of the tumor microenvironment, such as the extracellular matrix and matrix metalloproteinase [130]. In this regard, Mutter et al. analyzed the value of circulating tumor deoxyribonucleic acid (ctDNA) in predicting the outcome of CNS lymphomas. The presence of ctDNA in pretreatment plasma was significantly associated with unfavorable OS and PFS [131]. These aspects illustrate how prognostic assessment in PCNSL can be further refined in the future in order to achieve adequate prognostic scoring even in the context of T-cell-based immunotherapies and, in particular, to reflect the influence of the immune microenvironment.

Beyond tissue-, blood-, and CSF-related biomarkers, there are multiple suggestions of radiological patterns that could serve as potential surrogates for outcomes and potentially reflect local TME compositions. Recently, Kaulen et al. identified radiological risk factors for failure of CD19-directed CAR T-cell therapy in CNSL: In primary refractory disease, CNSL lesions with peripheral contrast enhancement patterns were significantly enriched and associated with a lower ORR [132]. Peripheral contrast enhancement patterns have been identified as a potential marker related to an immunosuppressive transcriptional cluster in cases of CNSL [81]. Further, baseline leptomeningeal disease was associated with an increased risk of early recurrence following an initial complete remission [132]. Chien et al. analyzed the diagnostic impact of apparent diffusion coefficient (ADC) characteristics derived from diffusion-weighted imaging (DWI). Lower ADC values and corresponding ratios were consistent with significantly lower PFS, indicating an early predictive value for r/r PCNSL [133]. These data reinforce the importance of tumor architectural and biological features reflected by DWI [134]. 

## Therapeutic implications and future directions

### T-cell–based immunotherapeutic strategies

**Fig. 5:**
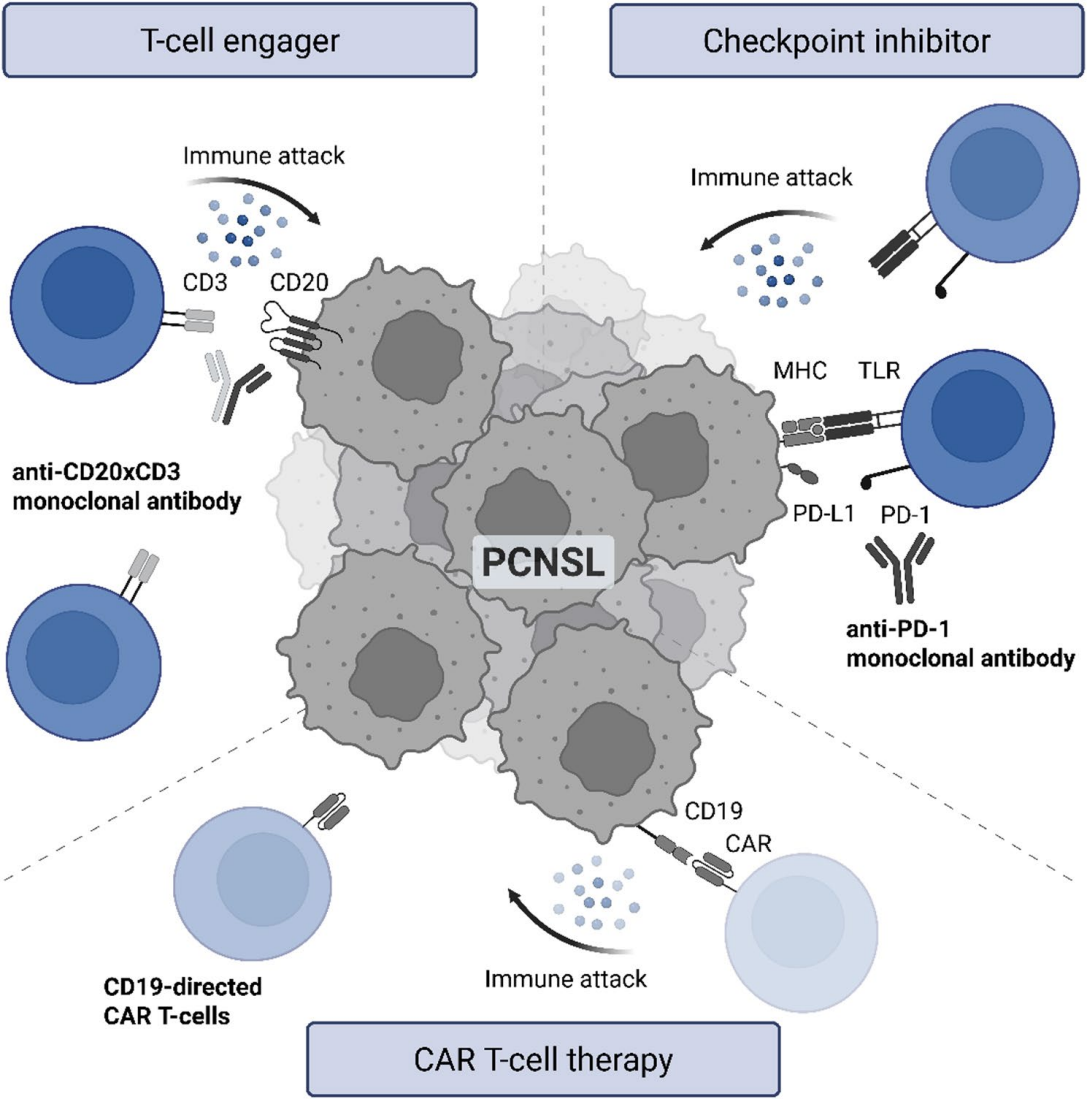
Therapeutic implications of T-cell based immunotherapies in PCNSL. The most prominent strategies include checkpoint inhibitors (e.g., nivolumab or pembrolizumab), T-cell engagers (e.g., glofitamab), and genetically engineering CAR T-cells (e.g., CD19-directed CAR T-cell therapy, axicabtagene ciloleucel or tisagenlecleucel). Created with *Biorender.com* (https://BioRender.com/wkoc587)

The immune-privileged nature of the CNS poses both immunological and therapeutic challenges in the treatment of PCNSL. Drug effectiveness strongly depends on their sufficient accumulation in the CNS. Therapy protocols incorporating HD-MTX as a central component have thus become the standard for induction treatment of eligible PCNSL patients, followed by consolidating HDC-ASCT [9, 10, 135, 136]. Although current treatment options for PCNSL achieve favorable outcomes in a substantial proportion of younger patients, the recurrence rate is high and recurrence treatment is challenging. Furthermore, older patients with reduced biological reserves have limited therapeutic options and generally poorer outcomes, highlighting a critical unmet medical need. T-cells are playing a key role in tumor immunosurveillance and numerical as well as functional competence have been linked to favorable treatment outcomes in malignant diseases, particularly in hematological entities [137, 138]. Nowadays, T-cell-based immunotherapeutic approaches have yielded promising results in a number of malignancies. The most prominent strategies include checkpoint inhibitors, T-cell engagers (TCE), and genetically engineered CAR T-cells [139]. While other immunotherapeutic strategies under investigation have been less successful in treating PCNSL, promising results have been especially obtained from CAR T-cells. Table 2 provides an overview of currently recruiting or planned clinical trials for PCNSL. In addition, the main mechanisms of action of these immunotherapies are illustrated in Fig. 5.

**Table 2:** Overview of currently recruiting or planned immunotherapeutic trials for PCNSL

Trial ID	Phase	Indication	Treatment	Status	Country
**Checkpoint inhibitors**					
NCT06475235	I	1st line PCNSL	pembrolizumab + R-MT	recruiting	USA
NCT04421560	I/II	r/r PCNSL	pembrolizumab + rituximab/ibrutinib	recruiting	USA
NCT05425654	II	1st line PCNSL	RL-MPV, followed by HDC-ASCT and nivolumab maintenance	recruiting	Russia
NCT04609046	I	1st line PCNSL	R-HD-MTX + nivolumab/ lenalidomide	recruiting	USA
NCT04401774	II	1st line PCNSL	nivolumab maintenance, if persistent CSF ctDNA after 1st line	not recruiting	USA
NCT04022980	I	1st line PCNSL	nivolumab consolidation in patients > 65 years	not recruiting	USA
NCT04961515	II	r/r PCNSL	sintilimab/orelabrutinib/ temozolomide	recruiting	China
NCT05253118	II	r/r CNSL	tislelizumab/pemetrexed	not recruiting	South Korea
**T-cell engagers**					
NCT06922604	I	r/r CNSL	glofitamab	recruiting	USA
NCT07082868	I	r/r CNSL	epcoritamab/ibrutinib	recruiting	USA
NCT06931652	II	r/r CNSL	epcoritamab/rituximab/ lenalidomide	not recruiting	France
**CAR T-cell therapy**					
NCT06876688	II	r/r PCNSL	relmacabtagene autoleucel (CD19-directed CAR T-cells) followed by tislelizumab	recruiting	China
NCT07198464	n/a	1st line and r/r PCNSL	relmacabtagene autoleucel (CD19-directed CAR T-cells) followed by HDC-ASCT, orelabrutinib, sintilimab	not recruiting	China
NCT05651178	I	r/r CNSL	CD19/CD22-directed CAR T-cells	recruiting	China
NCT06213636	I/II	r/r B-cell malignancies	CD19/CD22-directed CAR T-cells	recruiting	China
NCT04443829	I	r/r PCNSL	CD19-directed CAR T-cells	active, not recruiting	UK
NCT04532203	I	r/r B-cell malignancies	CD19-directed CAR T-cells	recruiting	China
NCT05625594	I	CNSL	ICV CD19-directed CAR T-cells	recruiting	USA
NCT04464200	I	r/r B-cell malignancies	CD19-directed CAR T-cells	recruiting	USA
NCT07062627	I	r/r CNSL	anbalcabtagene autoleucel (CD19-directed CAR T-cells)	recruiting	South Korea
NCT04186520	I/II	r/r B-cell malignancies	CD19/CD20-directed CAR T-cells	recruiting	USA
NCT07137494	I	r/r CNSL	zamtocabtagene autoleucel (CD19/CD20-directed CAR T-cells)	recruiting	USA
NCT04792489	II	r/r B-cell malignancies	zamtocabtagene autoleucel (CD19/CD20-directed CAR T-cells)	recruiting	USA

Abbreviations: ASCT - Autologous stem cell transplantation, CAR - Chimeric antigen receptor, CNSL - Central nervous system lymphoma, CSF - Cerebrospinal fluid, ctDNA - circulating tumor deoxyribonucleic acid, HDC - High-dose chemotherapy, ICV - Intracerebroventricular, MTX - Methotrexate, PCNSL - Primary central nervous system lymphoma, R-HD-MTX - Rituximab, high-dose methotrexate, RL-MPV - Rituximab, lenalidomide, methotrexate, procarbazine, vincristine, R-MT - Rituximab, methotrexate, temozolomide, r/r - recurrent/refractory

As previously outlined, analyses of the PCNSL immune microenvironment revealed increased expression of immune checkpoint receptors. Therapeutic targeting of these receptors thus appears promising [31, 140]. In a preclinical, immunocompetent murine CNSL model, treatment with anti-PD-1 antibodies resulted in complete remission in 50% of mice and increased intratumoral concentration of CD8+ T-cells [141]. These findings prompted the clinical investigation of PD-1 inhibitors, primarily nivolumab and pembrolizumab, in clinical settings [142–147]. Although several case reports and small case series demonstrated encouraging response rates, the majority of patients experienced early relapse, with a median PFS of only 1–3 months [143, 146]. The phase 2 CheckMate 647 trial (NCT02857426) showed disappointing results for nivolumab in relapsed/refractory (r/r) PCNSL and primary testicular lymphoma, with an overall response rate (ORR) of only 6.4% and a median PFS of 1.4 months in the PCNSL cohort. These findings suggest that anti-PD-1 monotherapy is insufficient to achieve durable disease control, which has led to the investigation of PD-1 inhibitors in combination with other agents, such as BTKi [148, 149], or with HD-MTX-based regimens. For example, sintilimab in combination with MTX, rituximab, and temozolomide was evaluated as a first-line treatment in patients with PCNSL. Among 27 patients included in the study, ORR was 96.3% after a median follow-up of 24.4 months [99]. Notably, such combinations of PD-1 blockade with anti-CD20 antibodies are particularly intriguing, as PD-1 expression on myeloid cells has been associated with impaired phagocytic function, suggesting that PD-1 inhibition may not only reinvigorate T-cell–mediated immunity but also restore macrophage-mediated antibody-dependent phagocytosis, thereby synergizing with rituximab.

T-cell-engaging strategies function by activating and spatially redirecting resident T-cells within the tumor microenvironment to exert cytotoxic activity against defined tumor antigens. Glofitamab, a CD20xCD3 TCE, has demonstrated a favorable response in r/r DLBCL, and is currently under investigation as a potential first-line treatment, with encouraging study results to date [150, 151]. In secondary CNS lymphoma (SCNSL), that represent malign lymphoma that is not limited to the CNS alone, glofitamab was shown to partially penetrate the blood-brain barrier. The analysis of Godfrey et al. demonstrated the stimulation of tumoral immune cell infiltration and clinical response following glofitamab therapy in a small series of heavily pretreated (including CAR T-cell therapy) patients with SCNSL [152]. These findings are corroborated by a few reports on the use of glofitamab in r/r PCNSL, which also demonstrated rapid remission [153, 154]. However, the blood-brain barrier poses certain challenges: achieving sufficient bioavailability is hampered by tight endothelial junctions and active efflux systems. Additionally, plasma half-lives and renal clearance influence therapeutic exposure since they limit steady-state CNS penetration [155, 156]. Restricted lymphocyte trafficking within the CNS as an immune-privileged site, and the immunosuppressive impact of the tumor microenvironment are crucial for the efficacy of immunotherapies, including TCE. Consequently, systemic dosing often fails to achieve therapeutic concentrations in the PCNSL microenvironment. Pharmacokinetic studies indicate that TCE have a volume of distribution similar to plasma volume, suggesting intravascular confinement and limited tissue extravasation [157]. The observed CNS penetration appears to be variable and is likely dependent on disruption of the blood-brain barrier by the tumor itself or tumor-associated inflammation, rather than on intrinsic transport. The analysis by Godfrey et al. revealed that the CSF concentration of glofitamab ranged from 0.1% to 0.4% of the peripheral blood concentration [152]. Yang et al. reported similar CSF/plasma ratios of glofitamab [158]. Clinical evidence in PCNSL remains limited as large-scale prospective trials are lacking, and the currently available data are promising but do not yet provide robust estimates of response rates.

The implementation of CAR T-cell therapies represented a revolutionary breakthrough in the treatment of systemic DLBCL. Potential challenges in CAR T-cell trafficking to the CNS and the risk of immune cell-associated neurotoxicity syndrome (ICANS), however, led to PCNSL patients being excluded from initial CD19-directed CAR T-cell trials examining the efficacy of axicabtagene ciloleucel and tisagenlecleucel in DLBCL [159, 160]. The use of CAR T-cell therapies in smaller case series of patients with PCNSL revealed promising feasibility, an acceptable safety profile and, above all, good efficacy [161, 162]. Furthermore, the prospective use of tisagenlecleucel was found to result in CAR T-cell trafficking into the CSF together with CAR T-cell expansion in the peripheral blood in PCNSL patients [163]. A retrospective analysis of overall 100 PCNSL patients treated with CAR T-cells (axicabtagene ciloleucel in 59%, tisagenlecleucel in 38% of cases) demonstrated an ORR of 60% at day 100 after CAR T-cell therapy. The two-year PFS and OS rates of the whole cohort were 28% and 37%, respectively [164]. Choquet et al. reported comparable results in a retrospective study, albeit with a shorter follow-up period [165]. A meta-analysis by Cook et al. suggests that around one-third of patients with PCNSL or SCNSL could achieve durable responses to CAR T-cell therapy [166]. Other approaches, such as CD19/CD22 dual-target CAR T-cell therapy following PD-1 and BTK inhibition as maintenance, also appear to be feasible [167]. Moreover, BTK inhibition has immunomodulatory effects, especially on T-cells, with the capacity to reverse T-cell exhaustion and enhance their antitumoral functions, as evidenced in CLL [168, 169]. Inhibiting BTK prior to T-cell apheresis has been demonstrated to enhance the yield and functionality of the CAR T-cell product and CAR T-cell persistence after reinfusion [170, 171]. BTK inhibition has also been investigated in PCNSL in a non-CAR T-cell therapy context and is currently recommended in the refractory setting, with particular suitability indicated for patients deemed unsuitable for intensive treatment approaches [1, 172–175]. Combining microenvironment-modulating immunotherapies in conjunction with or following CAR T-cell therapy has the potential to overcome resistance arising from an immunosuppressive tumor microenvironment by harnessing synergistic effects. Observations suggest that checkpoint inhibition may enhance the efficacy of CAR T-cells in different solid tumors [176–178]. In addition, a preclinical murine model reported by Brinkmann et al. demonstrated enhanced efficacy, improved expansion and persistence of CD19-directed CAR T-cells in combination with CD20xCD3 TCE therapy [179]. The replicability of these synergistic effects in PCNSL remains to be ascertained.

### Emerging concepts and unresolved challenges in PCNSL

In addition to the T-cell-based immunotherapeutic approaches that have been previously outlined, a number of other potential future therapeutic targets are currently under investigation (as recently reviewed by several groups) [180–185]. By linking lymphomagenesis and immune evasion, oncogenic signaling pathways demonstrate its influence on immunosuppression. Targeting these pathways holds therapeutic potential, given its capacity to intervene in the immunosuppressive microenvironment. In a murine model, the inhibition of the PI3K/AKT/mTOR signaling pathway using the dual PI3K/histone deacetylase inhibitor BEBT-908 was found to significantly inhibit the growth of induced brain tumors, resulting in improved survival outcomes [186]. Furthermore, targeting IRAK-4 with the oral, single-agent drug CA-4948 was found to reduce NF-κB expression in mice with PCNSL [187]. In future, AI-driven approaches could also contribute to precise PCNSL treatment, as demonstrated by AI-powered signaling pathway analysis [188]. These examples demonstrate the huge therapeutic potential of targeting oncogenic signaling pathways in PCNSL.

Despite major advances in the characterization of the immune microenvironment in PCNSL, several fundamental biological and translational questions remain unresolved. Recent spatial transcriptomic analyses demonstrated localized associations between malignant B-cell clusters and exhausted T-cell populations, suggesting pronounced intratumoral immune heterogeneity [31]. However, it remains unclear whether immune-rich and immune-poor tumor regions represent stable biological substructures or dynamically evolving during disease progression and therapy. Spatially resolved analyses may therefore help to determine whether local accumulation of M2-like macrophages, differential checkpoint ligand expression, or regional cytokine gradients actively establish physical and functional barriers that restrict T-cell infiltration and persistence within distinct tumor regions [30, 189]. In particular, future studies could clarify whether perivascular immune niches primarily support immune cell recruitment, whereas intratumoral niches preferentially promote T-cell exhaustion and immune escape. Furthermore, longitudinal spatial and multi-omic profiling approaches before and after immunotherapy may help to determine whether therapeutic pressure induces dynamic remodeling of tumor-immune interaction networks toward more immune-excluded microenvironmental states, as already demonstrated in other malignancies treated with immune checkpoint blockade [190]. 

Another unresolved issue concerns the mechanisms of resistance and local exhaustion following T-cell–based immunotherapies, particularly CAR T-cell therapy. While CD19-directed CAR T-cell approaches demonstrated encouraging clinical activity in PCNSL, durable remissions remain limited to a subset of patients [164–166]. Increasing evidence suggests that the CNS tumor microenvironment itself actively contributes to CAR T-cell dysfunction through several mechanisms. Persistent antigen exposure, expression of inhibitory immune checkpoint ligands, accumulation of M2-like TAMs, immunosuppressive cytokine release, metabolic competition, and restricted trafficking across the blood-brain barrier may collectively promote local T-cell exhaustion [28, 31–33, 91]. In addition, the CNS milieu itself may impose metabolic and immunological constraints on effector T-cells, including hypoxia, nutrient deprivation, and altered cytokine gradients. Strategies currently under investigation to overcome these barriers include dual-target CAR constructs, checkpoint inhibitor combinations, cytokine-enhanced CAR T-cells, and combination therapies incorporating BTKi or immune checkpoint blockade [167–171, 191]. 

Beyond therapeutic resistance, increasing attention has also been directed toward immune-mediated radiographic and neurological phenomena during immunotherapy. In CNS lymphoma, pseudoprogression and tumor inflammation-associated neurotoxicity (TIAN) may occur following immune activation and can complicate radiographic response assessment. Importantly, these phenomena may not merely represent treatment-related adverse events but could reflect local immune activation within the tumor microenvironment. Early clinical observations suggest that inflammatory radiographic changes following immunotherapy may correlate with enhanced anti-tumoral immune responses and favorable outcomes in selected patients [192]. However, distinguishing treatment-related immune activation from true disease progression remains a major diagnostic challenge in clinical practice. Advanced neuroimaging techniques, including DWI, perfusion imaging, and radiomic analyses, may therefore become increasingly relevant as surrogate markers of the local immune microenvironment. Recent studies indicate that radiographic characteristics may reflect distinct biological and immunological states of the CNS tumor microenvironment. In particular, peripherally contrast-enhancing lesions and leptomeningeal involvement have recently been associated with inferior treatment response and unfavorable outcome following CD19-directed CAR T-cell therapy in CNS lymphoma, potentially reflecting a more immune-suppressive tumor microenvironment with impaired immune cell trafficking and reduced local anti-tumoral activity [132]. In parallel, advanced neuroimaging parameters such as ADC characteristics derived from DWI have been linked to tumor cellularity, treatment response, and survival in PCNSL [193]. Taken together, these findings suggest that radiographic imaging patterns may serve as non-invasive surrogate markers of immune composition and functional microenvironmental states in CNS lymphoma, thereby complementing molecular and spatial immune profiling approaches.

## Conclusions

PCNSL is a rare and aggressive malignancy that arises within the unique immune-privileged environment of the CNS. Increasing evidence indicates that PCNSL biology is not solely dictated by tumor-intrinsic features, but is critically shaped by dynamic interactions between malignant B-cells and the local immune microenvironment. In this setting, the immune-restrictive nature of the CNS imposes distinct biological and therapeutic challenges, while simultaneously offering opportunities for immune-based interventions. A comprehensive understanding of the manner in which alterations in oncogenic signaling influence immune evasion strategies holds considerable potential for enhancing future therapeutic approaches in PCNSL. Recent advances have demonstrated encouraging clinical activity of both humoral and cellular immunotherapies, particularly T-cell–directed strategies, underscoring the therapeutic relevance of immune modulation in PCNSL. Given the profound prognostic and therapeutic implications of immune contexture, immune microenvironmental parameters should be increasingly integrated into future risk stratification models. Omics-driven and immune-informed prognostic frameworks hold promise to refine and complement established clinical scoring systems, thereby enhancing their accuracy and clinical utility. However, the successful translation of these approaches will depend on robust data infrastructure, real-world implementation, and prospective multicenter validation. Ultimately, integrating validated immune biomarkers with emerging immunotherapeutic strategies has the potential not only to improve individual patient outcomes but also to optimize the overall structure of care for patients with PCNSL.

## Data Availability

No datasets were generated or analysed during the current study.

## Abbreviations

3F score: Three-factor score
ADC: Apparent diffusion coefficient
AI: Artificial intelligence
ALC: Absolute lymphocyte count
αSMA: Alpha-smooth muscle actin
ASCT: Autologous stem cell transplantation
BCL-2: B-cell lymphoma 2
BCR: B-cell receptor
BTK: Bruton’s tyrosine kinase
CAR: Chimeric antigen receptor
CLL: Chronic lymphocytic leukemia
CNS: Central nervous system
CNSL: Central nervous system lymphoma
CSF: Cerebrospinal fluid
ctDNA: Circulating tumor deoxyribonucleic acid
DLBCL: Diffuse large B-cell lymphoma
DWI: Diffusion-weighted imaging
EBV: Epstein-Barr virus
ECOG: Eastern Cooperative Oncology Group
EFS: Event-free survival
FOXP3: Forkhead box P3
GABA: γ-aminobutyric acid
HDC: High-dose chemotherapy
HD-MTX: High-dose methotrexate
HLA: Human leukocyte antigen
ICANS: Immune cell-associated neurotoxicity syndrome
ICV: Intracerebroventricular
IELSG: International Extranodal Lymphoma Study Group
IFN-γ: Interferon-γ
IL-10: Interleukin-10
IPCG: International Primary CNS Lymphoma Collaborative Group
IRAK: Interleukin-1 receptor-associated kinases
JAK/STAT: Janus kinase/signal transducer and activator of transcription
KPS: Karnofsky performance status
LAG-3: Lymphocyte-activation gene 3
LBCL: Large B-cell lymphoma
LDH: Lactate dehydrogenase
MCD: MYD88/CD79B-mutated
miRNA: microRNA
MSKCC: Memorial Sloan-Kettering Cancer Center
mTOR: Mechanistic target of rapamycin
MTX: Methotrexate
MYD88: Myeloid differentiation primary response 88
NF-κB: Nuclear factor ‘kappa-light-chain-enhancer’ of activated B-cells
NHL: Non-Hodgkin’s lymphoma
NK cells: Natural killer cells
OCT4: Octamer-binding transcription factor 4
ORR: Overall response rate
OS: Overall survival
PCNSL: Primary central nervous system lymphoma
PFS: Progression-free survival
PI3K: Phosphatidylinositol 3-kinase
PD-1: Programmed cell death protein 1
PD-L1/2: Programmed death-ligand 1/2
PNI: Prognostic nutritional index
PS: Performance status
R-HD-MTX: Rituximab, high-dose methotrexate
RL-MPV: Rituximab, lenalidomide, methotrexate, procarbazine, vincristine
R-MT: Rituximab, methotrexate, temozolomide
RNA: Ribonucleic acid
r/r: recurrent/refractory
SCNSL: Secondary central nervous system lymphoma
SIRPα: Signal regulatory proteinα
sPD-L1/2: Soluble programmed death-ligand 1/2
SYK: Spleen tyrosine kinase
TAMs: Tumor-associated macrophages
TCE: T-cell engager
TIAN: Tumor inflammation-associated neurotoxicity
TILs: Tumor-infiltrating lymphocytes
TIM-3: T-cell immunoglobulin and mucin-domain containing-3
TLR: Toll-like receptor
Tregs: Regulatory T-cells
WBRT: Whole-brain radiotherapy
WHO: World Health Organization
